# Public-Private-People Partnerships (4P) for Improving the Response to COVID-19 in Iran

**DOI:** 10.1017/dmp.2020.202

**Published:** 2020-06-24

**Authors:** Hamed Seddighi, Sadegh Seddighi, Ibrahim Salmani, Mehrab Sharifi Sedeh

**Affiliations:** Student Research Committee, University of Social Welfare and Rehabilitation Sciences, Tehran, Iran; Department of Mechanical Engineering, K. N. Toosi University of Technology, Tehran, Iran; Chalmers University of Technology, Gothenburg, Sweden; Department of Health in Disaster and Emergency, School of Public Health, Shahid Sadoughi University of Medical Sciences, Yazd, Iran; Department of Disaster Public Health, School of Public Health, Tehran University of Medical Sciences (TUMS), Tehran, Iran

**Keywords:** COVID-19, infrastructure, Iran, Public–Private-People partnership (4P)

## Abstract

The Public–Private–People partnership (4P) is a significant element in disaster response. Coronavirus disease (COVID-19) as a pandemic has been the worst disaster in the last decades in Iran in terms of exposure and magnitude. In order to respond effectively, the Iranian Government needs an extra capacity, which may be provided by the private sector and people. This study aims to collect evidences of 4P pertaining to the COVID-19 response in Iran from February to April 2020. Partnership case studies are classified into 3 categories: (1) Public–private partnerships; (2) public–people partnerships; and (3) private–people partnerships. It was found that the Iranian Government has removed or diminished some of the barriers to cooperation. There was also more cooperation between the people, the private sector, and the public sector than during normal times (vs disasters). People participated in the response procedure through some associations or groups, such as religious and ethnic communities, as well as through non-governmental organizations. It has been shown that 4P is vital in disaster response and, in particular, to epidemics. The government can be more active in partnerships with the private sector and people in emergencies, such as the COVID-19 pandemic. Enhancing social capital, institutionalization, and developing required infrastructures by the government will improve public–private partnerships.

Iran announced the first confirmed cases of severe acute respiratory syndrome coronavirus 2 (SARS-CoV-2) infections on February 19, 2020.^1^ Iran has the 10th-highest number of coronavirus disease (COVID-19) cases in the world and has been the worst affected country in the Middle East until June 10, 2020.^2^ The latest statistics of COVID-19 in Iran on June 10 indicate that the total number of people living with COVID-19 in Iran reached 177 938, the death toll reached 8506, and the total number of people recovered from the disease, so far, reached 140 590.^2^

Outbreaks are considered natural disasters, according to the definition of the International Federation of Red Cross Red Crescent Societies.^3^ During disasters, the affected communities need extra capacities to be able to respond effectively.^4^ Capacity enhancement is one of the major challenges for the communities to which the public–private partnership (3P) seems to be a significant solution.^5^ The public–private partnership is a cooperative arrangement between 2 or more public and private sectors.^6,7^ To put it differently, it includes an agreement between a government agency and a non-governmental organization (NGO) to develop a better infrastructure or to raise the ability of the community to work effectively.^8^ In recent years, researchers introduced a new concept that combines 3P with society (people) to form a public–private–people partnership (4P).^9^ This proposed cooperation model was raised just after that previous concept (public–private sectors cooperation) had been criticized due to lack of transparency and non-participation of people in programs.^10^ Participation of people, along with the public and private sectors, can make the public sector accountable and also help reach an effective implementation.^11^ This study aims to investigate 4P in the COVID-19 response throughout Iran.

## CLASSIFICATIONS

**FIGURE 1:**
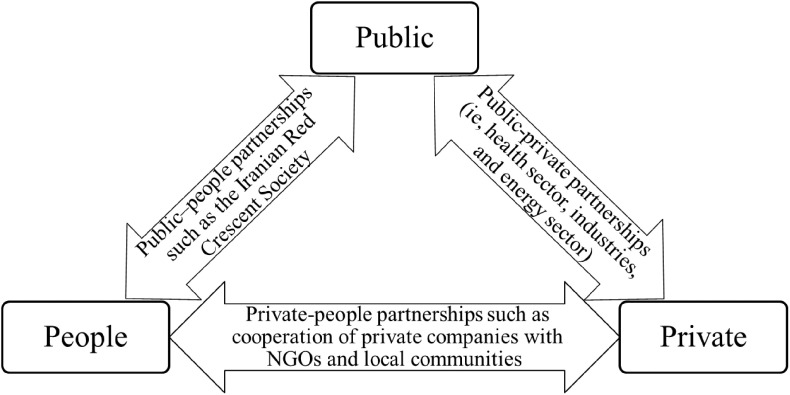
Public–Private–People Partnerships for the COVID-19 Response in Iran.

In this study, partnership case studies are classified into 3 categories: (1) public–private partnerships; (2) public–people partnerships; and (3) private–people partnerships. As shown in Figure 1, the present study presents some examples for each category in Iran.

### Public–Private Partnerships

Critical infrastructures are essential assets for the functioning of the society and economy. Major examples are energy, housing, agriculture, public health, transportation, telecommunication, and economic sectors. Infrastructures and the expertise to develop them are essential for any government. However, most often, such infrastructures belong to the private sector.^12^ Thus, in times of an emergency, a strong partnership is required between governments and private sectors to use such infrastructures.

The public–private partnership for critical infrastructure is the most popular partnership during the response to COVID-19 in Iran. For example, many private hospitals shared their facilities and equipment with the government to increase the capacity of the health care sector. In Iran, 20% of hospitals belong to the private sector and are doing their best to help the government.^13^ In addition, 117 new medical laboratories have been added to the current capacity of the health care sector during the period.^14^

In the telecommunication sector, there was a wide partnership between the Iranian Government and private companies. The major Iranian telecommunication companies, such as Mobile Telecommunication Company of Iran (MCI) and Irancell, developed their services, especially Internet service, during the COVID-19 crisis.^15,16^ People on lockdown need the Internet for remote working and remote education. These companies dedicated 100 gigabytes of free Internet to home users.^15,16^ According to the Telecommunication Infrastructure Company, the bandwidth of national telecommunication was improved significantly in response to the increasing usage of Internet-based services.^15,16^

In the production of goods related to the treatment and prevention of COVID-19, the government issued a permit to repurpose production lines and change them into dedicated lines for the production of alcohol, antiseptic products, and face masks without bureaucracy and as soon as possible (at least for 3 months).^17^ For further cooperation with private companies, the government decided to lift the import ban, impose export bans and restrictions, and authorize the Ministry of Industry, Mines and Trade to specify the import-export tariffs and duties for items required by the Ministry of Health (MOH).^17^ This has encouraged many companies to contribute to the country’s existing capacity in this regard. For example, the CEO of the Industrial Towns Company of Tehran Province announced that 40 production units in Tehran Province had changed their lines to produce health and medical materials necessary to fight against COVID-19 and 52 units had increased their production capacity.^18^ Similar partnerships have been implemented in other provinces of Iran.^18^

In recent years, Iran has been struggling with many challenges in providing technological equipment within the country.^19^ To overcome the challenge during the COVID-19 response, the Iranian Government cooperated with knowledge enterprises to produce its needs. Many knowledge-based companies are working under the umbrella of science and technology parks in different provinces for the COVID-19 response, and more than 400 knowledge enterprises have stepped in to produce COVID-19-related products.^20^ Such activities positively influence various fields of equipment and treatment. One instance is raising the ability of production units to produce approximately 1 million face masks per day, one and a half million liters of disinfectants per day, as well as ventilators, oxygen tanks, non-contact thermometers, protective clothing, and testing kits.^20^

In order to help the people who may have lost their income during the lockdown, the water, electricity, and gas companies have given a 3-month moratorium to the citizens for paying their outstanding bills.^21^ As the utility usage (water, electricity, and gas) was measured in Iran by an officer’s visit and it was associated with higher risks of COVID-19 outbreak, the utility companies allowed the customers to self-report their utility consumption during the pandemic.^21^ The utility companies also ensured that no customer would be penalized or subject to shutting off their utilities in this period for nonpayment of bills. In addition, the COVID-19 response has increased domestic water consumption by 40% compared with the same period in the last year.^22^ Thus, the Iranian Urban Water and Wastewater Management Company increased its capacity according to the consumption raised.^21^ To enable the production of face masks, gloves, and protective gear, the private petrochemical companies have focused on the production of initial and raw materials required for fighting against COVID-19, such as protective gear and gloves.^23^ For instance, the Karoun petrochemical company has focused on producing a large amount of bleach to be used for disinfection.^24^ Arvand petrochemical company has started to step up its caustic production used as raw material for hygiene products such as soaps.^24^ The private gas stations contributed to such efforts by providing full service so that every single car is filled up by a gas station attendant to reduce the transmission of the virus by infected drivers.^25^

During the response to COVID-19, Digikala (the biggest online shop in Iran^26^) has seen a 2.5-fold increase in online shopping demand compared to its forecast.^27^ According to the memorandum made between COVID-19 Response Headquarters and Digikala Company, the company will allow the 1000 businesses damaged during COVID-19 to present and sell their products on its marketplace platform free of charge and free of any commission fee.^28^ Costs for product processing, packaging, and delivery to the customer’s address all over the country shall be paid by Digikala.^28^ Furthermore, according to the memorandum, Digikala will provide such businesses with webinars for training how to use online shop infrastructures and other relevant education services.^27^ Some of the provisions of the agreed memorandum include granting the free online marketing credit and activating the online shop within 2 days, providing transportation logistics services throughout Iran, providing such businesses with 12 centers all over the country for receiving their products, and sending them to their customers around the country using Digikala’s shipping infrastructure.^28^ Due to the greater tendency of people to shop online, this platform has experienced a growth of nearly 70% in the company’s daily new clients.^27^ The company has also hired 700 new staff members and has the plan to hire 1300 more to meet the demand of its customers.^27^ Digikala has been able to increase the order registration capacity to over 500 000 items per day.^27^ This increased capacity was not exclusive to Digikala and many online shops have experienced such capacity.^29^

### Public–People Partnerships

The coordination between people (ie, civilians, NGOs, local communities, and faith-based organizations) with public and professional organizations is a determinative factor in the effectiveness of emergency response.^30-32^ During the COVID-19 epidemic in Iran, many public–people partnerships were formed around the country. For example, the Health Network (MOH office in counties) in Meybod County, Yazd Province, having the support of community leaders, including the county’s high ranking religious leader, formed a group to which one-third of the county families joined. The group was called “COVID-19 Response by Practicing Self-Care” aiming to serve people by raising the public awareness, donor management, psychosocial support, and helping the affected families.^33^ Another example is an NGO in the city of Yazd, called *Culture House*, which raised US $100 000 via donors in cooperation with governmental organizations and spent it to contribute to the local hospitals and emergency organizations by purchasing face masks, protective equipment, and livelihood kits for affected low-income families.^34^

Collaborative partnerships between the government and civil society have a long history throughout the world.^35^ This category covers a number of government-formed collaborations with organizations, such as international and regional NGOs, faith-based groups, state non-profit assistance agencies, and professional societies.^30^ The most prominent example in response to COVID-19 in Iran is the partnership of the government and the Iranian Red Crescent Society (IRCS). The IRCS is a member of the COVID-19 Response Headquarters in Iran. In the division of work among members, one of the tasks assigned by the headquarters to IRCS was establishing testing centers using the large number of volunteers.^36,37^ COVID-19 Testing Centers or mobile fever detection stations started work on March 18 in Iran.^37^ The COVID-19 screening plan has been conducted by the IRCS for 17 days with the aim of identifying and treating people with COVID-19, reducing road trips, and sensitizing people.^37^ In 851 screening posts, with the help of 95 371 volunteers, a total of 21 640 866 people were monitored by the IRCS.^38^ Furthermore, the IRCS supported the government by raising public awareness, providing psychosocial support, collecting donation, sharing health care facilities and ambulances, and helping with the livelihood of low-income families affected by COVID-19.^39^ Partnerships between the government and the IRCS to use the capacity of volunteers have a long history in Iran.^1,40,41^

### Private–People Partnerships

In the first days after the flood, Digikala (the biggest online shop in Iran^27^) started a campaign in collaboration with the IRCS to collect aid and send it to the provincial branch of the IRCS in Golestan.^26^ The IRCS listed all necessary requirements, and Digikala put listed products at discount on a special page of its website. People as customers could buy blankets, warm clothes, and hygiene kits from Digikala and send them directly to the IRCS branch in the affected area to be distributed.^26^

Many vulnerable groups during the COVID-19 crisis are in need of help due to the pandemic-associated recession. In order to help the vulnerable groups, Iranian giant distribution company of Digikala, in a cooperation with 4 NGOs engaged in helping homeless people and children,^42^ sells products at a 50% discount in favor of the mentioned NGOs. In such a procedure, any individual donor selects the desired NGO first. Then the person selects the desired goods and enters the announced discount code when the order is registered and, finally, grants the gift to the desired charity.^42^

## DISCUSSION

This study aims to present evidences on partnerships formed for the COVID-19 response in Iran. It has found that, in order to respond to the pandemic, 4P was developed. The cases presented in the study were classified into 3 different types, including public–private partnerships, public–people partnership, and private–people partnerships. Critical infrastructures provide the most important services, including water, energy, telecommunication, health, and transport. Usually, disasters adversely affect infrastructures. Although the disease has not directly affected the infrastructure, it has indirect influences. As demand increases, it leads to increased supply in the short term. Therefore, a special effort is needed. For example, the COVID-19 outbreak has dramatically increased the need for hospital beds, doctors, ventilators, and preventive equipment such as face masks. As the government were not able to increase public sector capacity within a short time, the need for private sector cooperation in order to improve the capacity to respond to this pandemic was strongly recognized. Due to the high prevalence of the virus, the public sector lacked sufficient capacity to inform, assist vulnerable people, and persuade the public. People participated in the response procedure through some associations or groups, such as religious and ethnic communities, as well as through NGOs including the IRCS. The IRCS, for example, participated in the screening task using the potential capacity of volunteers, and local communities contributed through persuading people to stay home. Different studies suggest that the contribution of communities and people in anticipating risks, limiting risk effects, and accelerating recovery after a disaster is vital.^43,44^ Of course, public sectors asking for help from private sectors and for people to respond does not necessarily indicate the inability of the government or lack of resources to respond. It sometimes reveals the difficulties faced by the government in allocating available resources,^44^ meaning that it fails to allocate the resources properly due to the inefficiency of the system.^44^ In addition, some studies have suggested that the government’s inattention to disaster management had been partially offset by greater public participation.^45-47^ There are different studies about 3P in Iran, especially about the health sector.^48,49^ Sadeghi et al. (2016) introduced 4 strategies to develop and promote 3P in the provision of hospital services in Iran, including modifying the policies and regulations, sociocultural changes, improvement of current mechanisms and processes, and financial and capital capacity building.^48^ Danaei et al. indicated 9 barriers to the development of 3P in Iran, including the following:

- Economic barriers (ie, bad economic and trade conditions in Iran)
- Political barriers (ie, cancellation of government agreements as a result of government change)
- Legal barriers (ie, lack of a clear legal framework)
- Structural barriers (ie, lack of specialized and professional institutions for 3P projects)
- Procedural barriers (ie, low capacity and skills of the public sector to manage public–private partnership projects)
- Strategic barriers (ie, government policy in providing infrastructure)
- Executive barriers (ie, complexity of tax status of public–private partnership projects)
- Human barriers (ie, poor understanding of politicians and decision-makers on 3P)
- Sociocultural barriers (ie, low motivation of the private sector in partnership with the public sector)^50^

It seems that the Iranian Government has tried to remove some of the abovementioned obstacles while responding to COVID-19 through several measures, such as reducing import duties, reducing bureaucracy to obtain the required industry licenses, and supporting knowledge-based companies fulfilling technology-based needs.

However, the government is required to consider some factors to increase 4P for a proper response to emergencies, including COVID-19. The significant element for a successful COVID-19 response is social capital (ie, trust). Social capital emphasizes the positive effects of social activities and acts as an important aspect of nonmonetary capital.^30^ Seddighi^1^ found that trust among the government, people, and NGOs is a major problem in the COVID-19 response in Iran, and trust is required to develop new policies to overcome mistrust. Further research is needed to survey the role of trust in 4P during COVID-19 in Iran. The other factor required for a successful 4P is a strong and suitable Internet infrastructure during the COVID-19 epidemic. Many efforts in different sectors, such as administration, education, and economy, need proper access to the Internet. The Internet penetration rate in Iran is 87%.^51^ However, many people connect to the Internet via their mobile data connection rather than home Internet.^52^ This seems to be a challenge in developing 4P. In order to coordinate informally, it is needed to institutionalize many of the routines and practices of a partnership. Chen (2013) found that the higher the degree of institutionalization, the greater the likelihood of partnerships being successful.^30^

## CONCLUSION

This study found that the Iranian Government has removed or diminished some of the barriers to cooperation. There was also more cooperation among the people, the private sector, and the public sector than during normal times (vs disasters). This study has shown that 4P is vital in disaster response and, particularly, during epidemics.

It could be concluded that the Iranian Government should be more active in arranging partnerships with private sectors in emergencies via official memorandums. Besides the health care sector, where the government was actively seeking the partnership with the private sector to increase the capacity for treatment and production of medical goods, in other areas, the private sector recognized the demand of the communities during the pandemic and increased its capacity accordingly. It can be suggested that, in disasters such as earthquakes, floods, and pandemics, there should be prior preparation for better interaction among the public sector, private sector, and people. Such preparation includes the conclusion of memorandum agreements between the public and private sectors, especially in the area of critical infrastructure and relief items. The people’s partnership through NGOs after disasters is vital. Therefore, the role of NGOs and local communities in response and rehabilitation must be determined in advance in order to have an effective and fast response.
